# Determinants of seropositivity among HPV-16/18 DNA positive young women

**DOI:** 10.1186/1471-2334-10-238

**Published:** 2010-08-11

**Authors:** Carolina Porras, Christina Bennett, Mahboobeh Safaeian, Sarah Coseo, Ana Cecilia Rodríguez, Paula González, Martha Hutchinson, Silvia Jiménez, Mark E Sherman, Sholom Wacholder, Diane Solomon, Leen-Jan van Doorn, Catherine Bougelet, Wim Quint, Mark Schiffman, Rolando Herrero, Allan Hildesheim

**Affiliations:** 1 Division of Cancer Epidemiology and Genetics, National Cancer Institute, Bethesda, MD, USA; 2 Proyecto Epidemiológico Guanacaste, Fundación INCIENSA, Liberia, Costa Rica; 3 Women and Infants' Hospital, Providence, Rhode Island; USA; 4 DDL Diagnostic Laboratory, Voorburg, The Netherlands; 5 GlaxoSmithKline Biologicals, Rixensart, Belgium

## Abstract

**Background:**

Not all women infected with HPV-16/18 have detectable levels of HPV-16/18 antibodies, those who seroconvert develop low antibody levels, and seroconversion occurs typically several months post-infection. We evaluated determinants of seropositivity among 646 women infected with HPV-16 and/or HPV-18.

**Methods:**

Data are from the enrollment visit of the NCI-sponsored Costa Rica HPV Vaccine Trial. Sera were tested for HPV-16/18 antibodies by ELISA; cervical specimens were tested for HPV DNA using HC2 and SPF_10_/LiPA_25_. Odds ratios (OR) and 95% confidence intervals (CI) were computed.

**Results:**

Among HPV-16/18 DNA positives, seropositivity was 63.0% and 57.5%, respectively. Among HPV-16 DNA positives, seropositivity increased with lifetime number of sexual partners (p-trend = 0.01). Women with abnormal cytology and/or high viral load had a 1.63-2.79-fold increase in the detection of antibodies compared to women with normal cytology/low viral load. Current users of oral contraceptives had a 1.88-fold (95%CI, 1.14-3.09) increased detection of antibodies and current users of injectables had a 3.38-fold (95%CI, 1.39-8.23) increased detection compared to never users. Among HPV-18 DNA positive women, seropositivity was associated with current oral contraceptive use (OR 2.47; 95%CI 1.08-5.65).

**Conclusions:**

Factors associated with sustained HPV exposure (abnormal cytology, elevated HPV viral load, increasing lifetime partners) were predictive of HPV-16 seropositivity. Hormonal contraceptive use was associated with seropositivity suggesting an effect of hormones on immune responses to HPV. Patterns were less consistent for HPV-18. Follow up of incident HPV infections to evaluate seroconversion and their determinants is needed.

## Background

Infections with most viruses typically result in rapid generation of antibodies that protect against re-infection. In contrast, not all women infected with human papillomavirus (HPV) 16/18 have detectable levels of anti HPV-16/18 antibodies. Women who seroconvert develop low antibody levels and seroconversion occurs within months and varies among women [1,2]. The slow and weak antibody response generated by HPV infections could be explained by its life-cycle in the host. HPV is shed within intact cells lining mucosal surfaces, which limits exposure of the host immune system to the virus. HPV infected cells that undergo lysis (i.e. koilocytes) are shed to the environment and infections do not produce viremia. Finally, infections produce a limited load of HPV antigenic proteins [3,4].

Several studies have shown that, as expected, sexual behavior is the strongest predictor of HPV-16/18 antibody detection [5-7]. Other factors identified less consistently (smoking [8,9], oral contraceptive use [7], and history of other sexual transmitted infections [10]) could represent residual confounding by sexual behavior.

In contrast to studies that have evaluated the overall determinants of HPV-16/18 seropositivity, which cannot distinguish risk factors for exposure/infection from those associated with seroconversion given infection, little is known about the specific predictors of seropositivity given concomitant infection with cervical HPV-16 or HPV-18. To better understand why some women with cervical HPV-16/18 infection have detectable levels of antibodies while others do not, using data from a community-based HPV-16/18 vaccine trial of 7,466 women aged 18-25 years in Costa Rica, we analyzed the determinants of HPV-16 and18 seropositivity among the subset of 646 women found to be infected with cervical HPV-16 and/or HPV-18 at enrollment.

## Methods

### Study population

Data are from the enrollment (pre-vaccination) visit of a trial investigating efficacy of an HPV-16/18 vaccine to prevent infections and cervical neoplasia in the provinces of Guanacaste and Puntarenas, Costa Rica. The study design and procedures have been described in detail [11]. Briefly, women were identified through a population census and women 18 to 25 years old were invited to participate between June 2004-December 2005. A total of 7,466, approximately 60% of eligible women (30.5% of the census), agreed to participate and fulfilled the inclusion criteria. Eligible women were not hysterectomized, pregnant or lactating, were mentally competent, in good general health, willing to use a contraceptive method during the vaccination phase. Women with a history of chronic or immunodeficiency conditions or with a history of hepatitis A infection or vaccination against it, were excluded.

### Study procedures

At the clinic women gave informed consent and were interviewed. Information regarding sociodemographic factors, reproductive and sexual history, contraceptive use, and smoking were obtained. A physician obtained the medical history and performed a physical exam including a pelvic exam among sexually experienced women. At the pelvic exam, exfoliated cervical cells were collected using a Cervex brush and were rinsed into a vial containing 20 mL of PreservCyt solution. Samples were transported to the laboratory, where two 0.5 ml aliquots were drawn for HPV DNA testing by PCR. ThinPrep slides were prepared for cytology, and the remaining solution was used for HPV DNA detection by Hybrid Capture 2 (HC2). A blood sample was collected from all participants using a 10 ml vacutainer tube without additive. At the local laboratory serum aliquots for the determination of HPV-16 and HPV-18 antibodies by ELISA were obtained and frozen immediately.

Protocols were approved by the institutional review boards of INCIENSA, Costa Rica and National Cancer Institute, United States.

### HPV DNA detection and genotyping by SPF_10_/DEIA/LiPA_25 _system

HPV DNA detection and genotyping was performed at DDL Diagnostic Laboratory (DDL, Voorburg, The Netherlands) using PCR amplification with SPF_10 _primers followed by DNA enzyme immunoassay detection of amplimers. HPV typing on positive amplimers was performed using line probe assay (LiPA_25_) (Labo Bio-medical Products, Rijswijk, Netherlands), as previously described [12].

Since the trial uses a bivalent HPV-16/18 vaccine to maximize sensitivity for these types, all specimens that tested positive by SPF_10 _DEIA but negative for HPV-16 or HPV-18 by LiPA_25 _were tested for the presence of HPV-16 and HPV-18 using type-specific PCR primers set, as previously described [13,14].

The results of this assay were used to identify the group of women HPV-16 DNA positive or HPV-18 DNA positive at the enrollment visit of the vaccine trial.

### HPV DNA detection by Hybrid Capture 2

HC2 is a nucleic acid hybridization assay with signal amplification that combines antibody capture of DNA and RNA probe hybrids and chemiluminescent signal detection. The HPV HC2 test is designed to detect 13 carcinogenic HPV types (types 16, 18, 31, 33, 35, 39, 45, 51, 52, 56, 58, 59, and 68) without distinguishing the HPV type present. The test was performed according to the manufacturer's instructions in the laboratory at the University of Costa Rica.

With each assay a cut-off RLU (relative light units) value is calculated as the mean RLU value of three positive calibrators. Specimens with relative light units/cutoff (RLU/CO) value < 1.00 were considered negative; specimens with RLU/CO value ≥1.00 were considered positive for one or more of the carcinogenic HPV types detected by the test. Given that the magnitude of the RLU value above the cutoff of the assay is indicative of the total amount of high risk HPV type present, the results of this assay were used as a surrogate for HPV viral load.

### Determination of antibodies against HPV-16/18 L1 VLPs by ELISA

An enzyme-linked immunosorbent assay (ELISA) was used for the detection and quantitative determination of IgG antibodies against HPV-16 or 18 in serum specimens. HPV-16 and HPV-18 antibodies were measured separately; the assay was performed by GlaxoSmithKline Biologicals as described previously [15]. Briefly, 96 polystyrene well plates (MaxiSorp, Nunc) were coated at ~4° Celsius with VLP-16 (2.7 μg/mL) or VLP-18 (2.7 μg/ml) which were produced in a baculovirus expression system. After incubation and washing steps, plates were blocked with PBS containing 4% skim milk with 0.2% Tween 20. Samples were serially diluted in the blocking solution starting at 1/100 in two-fold increments. Serial dilutions of samples, standard, and controls were added to the microtiter plates. After incubation and washing, a peroxidase-conjugated anti-human polyclonal antibody was added to each well. Following incubation and washing the substrate and chromogen were added. The reaction was stopped and the intensity of the yellow color obtained was measured at 450/620 nm. Antibody levels, expressed as ELISA units/mL (EU/mL), were calculated by interpolation of OD values from the standard curve and by averaging the calculated concentrations obtained in a defined region of the curve. The assay cut-off was defined to be a value above three standard deviations of the geometric mean titer taken from a group of HPV-negative individuals. Seropositivity was defined as a titer greater than or equal to the assay cutoff established as 8 EU/mL for HPV-16 and 7 EU/mL for HPV-18 [16].

### Statistical Analysis

Of the 5871 women that at the enrollment visit had a pelvic exam, 8.3% (488) were HPV-16 DNA positive, 3.2% (188) were HPV-18 DNA positive, 0.5% (30) were HPV-16 and 18 positive. We restricted our analysis to the 484 women HPV-16 DNA positive and the 179 women HPV-18 DNA positive with available ELISA results. Additional information regarding the seroprevalence and determinants of HPV16/18 seropositivity for the total population was evaluated in a separate report [17].

Socio-demographic, sexual behavior, contraceptives, smoking and reproductive history characteristics, and cytology and HPV HC2 test results were evaluated as possible determinants of serological response.

For each characteristic, we calculated unadjusted odds ratios (ORs) for seropositivity and 95% confidence interval (CIs) using unconditional logistic regression. Of particular interest were variables that could be markers of timing of HPV infection (time since sexual debut and time with most recent partner) or of amount/load of exposure (number of sexual partners, viral load by HC2, cytologic finding, hormonal contraception, and condom use). Possible confounding factors were explored and a final model was built for each characteristic of interest adjusting for all other variables that changed the crude OR estimates by 15% or more. The outcome variables were seropositivity for antibodies against HPV-16 and seropositivity for antibodies against HPV-18.

We performed a similar set of analyses restricted to women with single HPV-16 and single HPV-18 infections by DNA testing. Results were comparable to those seen overall (data not shown).

From the HC2 test we used the ratio of relative light unit values to positive control (RLU/CO) as a surrogate for HPV viral load. Women were classified as having low or high viral load based on the median RLU/CO value observed (RLU/CO ≤ 30 were considered low viral load and RLU/CO > 30 were considered high viral load).

We assessed dose response associations (p-trend) by treating ordinal variables as continuous assuming a linear trend in the models. F test for differences in geometric means were calculated using ANOVA. Analyses were performed using Stata 10.0.

## Results

Among women who were HPV-16 DNA positive, seropositivity for antibodies against HPV-16 was 63.0% (305/484); among HPV-18 DNA positive the seropositivity for antibodies against HPV-18 was 57.5% (103/179).

### Determinants of seropositivity among HPV-16 DNA positive women

As shown in table 1, which presents unadjusted and adjusted risk estimates, there were no differences in the detection of anti-HPV16 antibodies by age (p-trend = 0.73). Among the demographic and sexual behavior factors evaluated, increasing lifetime number of sexual partner was significantly associated with seropositivity. After adjustment for time with most recent partner, women with three or more sexual partners in their lifetime had a 2-fold increase in the detection of anti-HPV16 antibodies compared to women with one lifetime sexual partner (p-trend = 0.01). Increasing frequency of sexual intercourse was indicative of increasing seropositivity; however it was not significant after adjustment for hormonal contraceptives (p-trend = 0.20).

**Table 1:** Determinants of seropositivity among HPV-16 DNA positive women at enrollment; Demographic and sexual factors.

Women characteristics	Anti-HPV16			Unadjusted		**Final model**^**1**^	
					
	Negative	Positive	%	OR	(95% CI)	OR	(95% CI)
**All women**	179	305	63.0				
**Age**							
18-19	50	84	62.7	1.00		1.00	
20-21	58	79	57.7	0.81	(0.50-1.32)	0.74	(0.44-1.25)
22-23	37	71	65.7	1.14	(0.67-1.94)	0.98	(0.51-1.86)
24-25	34	71	67.6	1.24	(0.73-2.13)	1.04	(0.53-2.06)
p trend					0.27		0.73
**Age at sexual debut**							
≥ 18	53	83	61.0	1.00		1.00	
17	33	63	65.6	1.22	(0.71-2.10)	1.22	(0.71-2.10)
16	37	60	61.9	1.04	(0.61-1.77)	1.04	(0.61-1.77)
15	34	57	62.6	1.07	(0.62-1.85)	1.07	(0.62-1.85)
< 15	22	42	65.6	1.22	(0.66-2.27)	1.22	(0.66-2.27)
p trend					0.69		0.69
**Years since first sexual intercourse**							
≤ 1	23	27	54.0	1.00		1.00	
2-3	45	75	62.5	1.42	(0.73-2.77)	1.28	(0.65-2.55)
4-5	45	74	62.2	1.40	(0.72-2.73)	1.23	(0.62-2.46)
≥ 6	66	129	66.2	1.66	(0.89-3.13)	1.40	(0.72-2.73)
p trend					0.15		0.39
**Frequency sexual intercourse, month**							
≤ 1	46	48	51.1	1.00		1.00	
2-3	26	52	66.7	1.92	(1.03-3.57)	1.85	(0.98-3.46)
4-9	59	110	65.1	1.79	(1.07-2.99)	1.55	(0.91-2.65)
≥ 10	45	92	67.1	1.96	(1.14-3.36)	1.57	(0.89-2.77)
p trend					0.03		0.20
**Lifetime number of sexual partners**							
1	49	63	56.3	1.00		1.00	
2	58	92	61.3	1.23	(0.75-2.03)	1.48	(0.87-2.50)
≥ 3	72	150	67.6	1.62	(1.02-2.59)	1.96	(1.19-3.25)
p trend					0.04		0.01
**Time with most recent partner, months**							
< 4	52	69	57.0	1.00		1.00	
4-13	56	98	63.6	1.32	(0.81-2.15)	1.24	(0.75-2.05)
> 13	71	138	66.0	1.46	(0.92-2.32)	1.54	(0.92-2.58)
p trend					0.11		0.10

**NOTE**. HPV, human papillomavirus; OR, odds ratio; CI, confidence interval.

^1 ^For each variable considered the final model adjusts for all variables that changed the crude estimate of risk by 15% or more.

Age: was adjusted for years since first sexual intercourse.

Age at sexual debut: was not adjusted for any co-factors since none of the potential confounders evaluated changed the OR estimates by 15% or more.

Years since first sexual intercourse: was adjusted for use of hormonal contraceptives.

Frequency sexual intercourse, month: was adjusted for use of hormonal contraceptives.

Lifetime number of sexual partners: was adjusted for time with most recent partner.

Time with most recent partner: was adjusted for lifetime number of sexual partners and use of hormonal contraceptives.

Age at sexual debut, years since first sexual intercourse and time with most recent partner were not significantly associated with detection of anti-HPV16 antibodies, although suggestive effect was observed for time with most recent partner (OR 1.54; 95% CI 0.92-2.58 for > 13 months versus < 4 months; p-trend = 0.10). Other factors such as years of education and marital status were not associated with seropositivity (data not shown).

Current use of hormonal contraceptives (oral contraceptive pill or injectable) was associated with seropositivity (Table 2). Women who reported use of oral contraceptives during the last month had a 1.88-fold (95% CI 1.14-3.09) increase in the detection of anti-HPV16 antibodies and current users of injectables had a 3.38-fold (95% CI 1.39-8.23) increase in the detection of anti-HPV16 antibodies compared to never users of hormonal contraceptives. We observed no significant association between condom use and anti-HPV16 antibodies, although there was suggestive evidence for reduced seropositivity among women who reported use of condom at the time of their last sexual intercourse (OR 0.66; 95% CI 0.42-1.03). To ensure that the higher seropositivity among hormonal contraception users was not confounded by condom use, we evaluated seropositivity and hormonal contraception for all strata of condom use (never, past, and current). We observed that, current users of hormonal contraceptives were more likely to be seropositive compared with never users of hormonal contraceptives in all strata of condom use. Current users of hormonal contraceptives who reported never using condoms had an OR of 2.20 (95% CI 0.83-5.81) and current users of hormonal contraceptives who reported current use of condom had an OR of 1.90 (95% CI 0.65-5.51) relative to condom users who did not use hormonal contraception.

**Table 2:** Determinants of seropositivity among HPV-16 DNA positive women at enrollment; Contraceptive, pregnancy, smoking status factors.

Women characteristics	Anti-HPV16			Unadjusted		**Final model**^**1**^	
					
	Negative	Positive	%	OR	(95% CI)	OR	(95% CI)
**All women**	179	305	63.0				
**Use of hormonal contraceptives**							
Never	42	45	51.7	1.00		1.00	
In the past	49	70	58.8	1.33	(0.76-2.33)	1.33	(0.76-2.33)
Current oral contraceptives	80	161	66.8	1.88	(1.14-3.09)	1.88	(1.14-3.09)
Current injectable	8	29	78.4	3.38	(1.39-8.23)	3.38	(1.39-8.23)
**Use of condom**							
Never	58	108	65.1	1.00		1.00	
In the past	74	143	65.9	1.04	(0.68-1.59)	1.12	(0.72-1.73)
Current (last month)	47	54	53.5	0.62	(0.37-1.02)	0.79	(0.46-1.37)
**Frequency of condom use**							
Never or rarely	67	128	65.6	1.00		1.00	
Sometimes	30	47	61.0	0.82	(0.48-1.41)	0.91	(0.52-1.59)
Most of the time	32	52	61.9	0.85	(0.50-1.45)	1.04	(0.60-1.81)
Always	46	78	62.9	0.89	(0.56-1.42)	1.01	(0.62-1.65)
**Use of condom last sexual intercourse**							
No	109	226	67.5	1.00		1.00	
Yes	70	79	53.0	0.54	(0.37-0.81)	0.66	(0.42-1.03)
**Number of pregnancies**							
0	81	109	57.4	1.00		1.00	
1	63	125	66.5	1.47	(0.97-2.24)	1.47	(0.97-2.24)
2	24	43	64.2	1.33	(0.75-2.37)	1.33	(0.75-2.37)
≥ 3	11	28	71.8	1.89	(0.89-4.02)	1.89	(0.89-4.02)
p trend					0.06		0.06
**Smoking Status**							
Never smoked for ≥ 6 months	140	231	62.3	1.00		1.00	
Former smoker	15	24	61.5	0.97	(0.49-1.91)	0.97	(0.49-1.91)
Current	24	50	67.6	1.26	(0.74-2.15)	1.26	(0.74-2.15)
**Cytology-Hybrid capture, viral load**^**2**^							
Normal/Low viral load	89	102	53.4	1.00		1.00	
LSIL/Low viral load	7	17	70.8	2.12	(0.84-5.34)	2.12	(0.84-5.34)
HSIL/Low viral load	5	16	76.2	2.79	(0.98-7.93)	2.79	(0.98-7.93)
Normal/High viral load	24	61	71.8	2.22	(1.28-3.85)	2.22	(1.28-3.85)
LSIL/High viral load	31	58	65.2	1.63	(0.97-2.75)	1.63	(0.97-2.75)
HSIL/High viral load	15	43	75.1	2.50	(1.30-4.81)	2.50	(1.30-4.81)

**NOTE**. HPV, human papillomavirus; OR, odds ratio; CI, confidence interval; LSIL, low grade squamous intraepithelial lesion; HSIL, high grade squamous intraepithelial lesion.

^1 ^For each variable considered the final model adjusts for all variables that changed the crude estimate of risk by 15% or more.

Use of hormonal contraceptives: was not adjusted for any co-factors since none of the potential confounders evaluated changed the OR estimates by 15% or more.

Use of condom: was adjusted for use of hormonal contraceptives.

Frequency of condom use: was adjusted for use of hormonal contraceptives.

Use of condom last sexual intercourse: was adjusted for use of hormonal contraceptives.

Number of pregnancies, smoking status and cytology-hybrid capture, viral load: were not adjusted for any co-factors since none of the potential confounders evaluated changed the OR estimates by 15% or more.

^2 ^Low viral load defined as hybrid capture rlu/co ≤ 30; High viral load defined as hybrid capture rlu/co > 30.

Number of pregnancies and smoking were not significantly associated with anti-HPV16 seropositivity (Table 2), although there was a tendency for seropositivity to increase with increasing number of pregnancies (OR 1.89; 95% CI 0.89-4.02 for ≥ 3 pregnancies compared to none; p-trend = 0.06).

Cytology and HC2 viral load measures were significantly associated with anti-HPV16 seropositivity (Table 2). Compared to women with a normal cytology and low viral load, those with evidence of SIL (squamous intraepithelial lesion) and/or high viral load had a 1.63 to 2.79 fold increase in the detection of anti-HPV16 antibodies.

Since previous studies have suggested that HPV is responsive to hormonal factors and that hormone levels modulate HPV viral expression [18,19], we examined whether the association between hormonal contraceptive use and anti-HPV16 seropositivity might be explained by elevated viral load among hormonal contraceptive users. Table 3 shows the analysis that compared the geometric mean (GM) for HC2 viral load levels by hormonal contraceptive status. We found no evidence that current hormonal contraceptives users had higher viral loads than never users (p = 0.66).

**Table 3:** Geometric mean for viral load (Hybrid capture, rlu/co values) stratified by use of hormonal contraceptives; **NOTE**. rlu/co, relative light units/cutoff.

Women characteristics	Geometric mean of Hybrid capture rlu/co values	**p**^**1**^
**Use of hormonal contraceptives**		
Never	40.0	
In the past	25.8	
Current oral contraceptives	30.1	
Current injectable	25.9	0.66

^1 ^F test for differences in mean log transformed hybrid capture rlu/co values.

Among women with antibodies against HPV16 we calculated the GM for anti-HPV16 levels for each category of all the variables evaluated and we did not find significant differences in serological levels, except for cytology-HC2 viral load (p = 0.003). Antibody levels were lowest among women with normal cytology and low viral load (GM = 50.0); intermediate among women with either abnormal cytology or high viral load (GM = 71.5); and highest among women with both an abnormal cytology and high viral load (women LSIL/high viral load, GM = 121.1; women HSIL/high viral load, GM = 97.7).

### Determinants of seropositivity among HPV-18 DNA positive women

Analyses that parallel those reported above for HPV-16 were performed to evaluate possible determinants of anti-HPV18 seropositivity among HPV-18 DNA positive women. Results are summarized in Tables 4 and 5. Women who reported having sexual intercourse 2-3 times a month were less likely to be seropositive (compared to women having intercourse less than twice a month); however this finding may be spurious since few women fell into this category. Compared to never users of hormonal contraceptives, current users of oral contraceptives but not injectables had increased detection of anti-HPV18 antibodies (OR 2.47, 95% CI 1.08-5.65). Also, when we evaluated antibody levels among anti-HPV-18 seropositive women by strata of hormonal contraceptives, we found that current users of oral contraceptives had significantly higher levels of anti-HPV18 (GM = 49.0 for never users, 30.4 for users in the past, 73.2 for current oral contraceptives users, 36.4 for current injectable users; p = 0.04).

**Table 4:** Determinants of seropositivity among HPV-18 DNA positive women at enrollment; Demographic and sexual factors.

Women characteristics	Anti-HPV18			Unadjusted		**Final model**^**1**^	
					
	Negative	Positive	%	OR	(95% CI)	OR	(95% CI)
**All women**	76	103	57.5				
**Age**							
18-19	17	27	61.4	1.00		1.00	
20-21	25	31	55.4	0.78	(0.35-1.74)	0.83	(0.36-1.91)
22-23	21	24	53.3	0.72	(0.31-1.67)	0.85	(0.30-2.40)
24-25	13	21	61.8	1.02	(0.41-2.55)	1.23	(0.39-3.85)
p trend					0.92		0.73
**Age at sexual debut**							
≥ 18	24	21	46.7	1.00		1.00	
17	13	26	66.7	2.29	(0.94-5.55)	2.29	(0.94-5.55)
16	12	21	63.6	2.00	(0.80-5.02)	2.00	(0.80-5.02)
15	17	14	45.2	0.94	(0.38-2.36)	0.94	(0.38-2.36)
< 15	10	21	67.7	2.40	(0.92-6.23)	2.40	(0.92-6.23)
p trend					0.36		0.36
**Years since first sexual intercourse**							
≤ 1	9	11	55.0	1.00		1.00	
2-3	18	30	62.5	1.36	(0.47-3.92)	1.25	(0.40-3.93)
4-5	18	23	56.1	1.05	(0.36-3.06)	0.87	(0.28-2.75)
≥ 6	31	39	55.7	1.03	(0.38-2.80)	0.77	(0.25-2.41)
p trend					0.70		0.32
**Frequency sexual intercourse, month**							
≤ 1	17	21	55.3	1.00		1.00	
2-3	18	7	28.0	0.31	(0.11-0.93)	0.25	(0.08-0.78)
4-9	24	35	59.3	1.18	(0.52-2.69)	0.97	(0.39-2.39)
≥ 10	16	39	70.9	1.97	(0.83-4.68)	1.47	(0.57-3.81)
p trend					0.03		0.13
**Lifetime number of sexual partners**							
1	20	28	58.3	1.00		1.00	
2	18	22	55.0	0.87	(0.37-2.04)	0.89	(0.37-2.15)
≥ 3	38	53	58.2	1.00	(0.49-2.02)	1.02	(0.48-2.17)
p trend					0.96		0.91
**Time with most recent partner, months**							
< 4	18	24	57.1	1.00		1.00	
4-13	26	34	56.7	0.98	(0.44-2.17)	0.81	(0.35-1.91)
> 13	32	45	58.4	1.05	(0.49-2.26)	0.84	(0.35-2.04)
p trend					0.87		0.74

**NOTE**. HPV, human papillomavirus; OR, odds ratio; CI, confidence interval.

^1 ^For each variable considered the final model adjusts for all variables that changed the crude estimate of risk by 15% or more.

Age: was adjusted for years since first sexual intercourse.

Age at sexual debut: was not adjusted for any co-factors since none of the potential confounders evaluated changed the OR estimates by 15% or more.

Years since first sexual intercourse: was adjusted for use of hormonal contraceptives.

Frequency sexual intercourse, month: was adjusted for use of hormonal contraceptives.

Lifetime number of sexual partners: was adjusted for time with most recent partner.

Time with most recent partner: was adjusted for lifetime number of sexual partners and use of hormonal contraceptives.

**Table 5:** Determinants of seropositivity among HPV-18 DNA positive women; Contraceptive, pregnancy, smoking status factors.

Women characteristics	Anti-HPV18			Unadjusted		**Final model**^**1**^	
	Negative	Positive	%	OR	(95% CI)	OR	(95% CI)
**All women**	76	103	57.5				
**Use of hormonal contraceptives**							
Never	17	14	45.2	1.00		1.00	
In the past	19	18	48.7	1.15	(0.44-3.00)	1.15	(0.44-3.00)
Current oral contraceptives	31	63	67.0	2.47	(1.08-5.65)	2.47	(1.08-5.65)
Current injectable	9	8	47.1	1.08	(0.33-3.53)	1.08	(0.33-3.53)
**Use of condom**							
Never	27	41	60.3	1.00		1.00	
In the past	33	40	54.8	0.80	(0.41-1.56)	0.87	(0.43-1.74)
Current (last month)	16	22	57.9	0.91	(0.40-2.03)	1.19	(0.51-2.80)
**Frequency of condom use**							
Never or rarely	31	50	61.7	1.00		1.00	
Sometimes	10	13	56.5	0.81	(0.32-2.06)	0.80	(0.31-2.11)
Most of the time	15	18	54.6	0.74	(0.33-1.69)	0.93	(0.40-2.17)
Always	19	22	53.7	0.72	(0.34-1.53)	0.80	(0.37-1.76)
**Use of condom last sexual intercourse**							
No	54	75	58.1	1.00		1.00	
Yes	22	28	56.0	0.92	(0.47-1.77)	1.23	(0.60-2.56)
**Number of pregnancies**							
0	38	44	53.7	1.00		1.00	
1	25	39	60.9	1.35	(0.69-2.62)	1.35	(0.69-2.62)
2	11	13	54.2	1.02	(0.41-2.54)	1.02	(0.41-2.54)
≥ 3	2	7	77.8	3.02	(0.59-15.43)	3.02	(0.59-15.43)
p trend					0.29		0.29
**Smoking Status**							
Never smoked for ≥ 6 months	56	75	57.3	1.00		1.00	
Former smoker	3	8	72.7	1.99	(0.51-7.85)	1.99	(0.51-7.85)
Current	17	20	54.1	0.88	(0.42-1.83)	0.88	(0.42-1.83)
**Cytology-Hybrid capture, viral load**^**2**^							
Normal/Low viral load	34	45	57.0	1.00		1.00	
LSIL-HSIL/Low viral load	4	8	66.7	1.51	(0.42-5.44)	1.51	(0.42-5.44)
Normal/High viral load	12	11	47.8	0.69	(0.27-1.76)	0.69	(0.27-1.76)
LSIL-HSIL/High viral load	24	36	60.0	1.13	(0.57-2.24)	1.13	(0.57-2.24)

**NOTE**. OR, odds ratio; CI, confidence interval; LSIL, low grade squamous intraepithelial lesion; HSIL, high grade squamous intraepithelial lesion; HPV, human papillomavirus.

^1 ^For each variable considered the final model adjusts for all variables that changed the crude estimate of risk by 15% or more.

Use of hormonal contraceptives: was not adjusted for any co-factors since none of the potential confounders evaluated changed the OR estimates by 15% or more.

Use of condom: was adjusted for use of hormonal contraceptives.

Frequency of condom use: was adjusted for use of hormonal contraceptives.

Use of condom last sexual intercourse: was adjusted for use of hormonal contraceptives.

Number of pregnancies, smoking status and cytology-hybrid capture, viral load: were not adjusted for any co-factors since none of the potential confounders evaluated changed the OR estimates by 15% or more.

^2 ^Low viral load defined as hybrid capture RLU/CO ≤ 30; High viral load defined as hybrid capture RLU/CO > 30.

## Discussion

We assessed the prevalence and determinants of serological response to HPV-16 and HPV-18 infections in a group of 646 women 18-25 years of age with concomitant detection of HPV-16 and/or HPV-18 at the cervix. One strength of this analysis is that unlike previous studies that evaluated determinants of anti-HPV seropositivity irrespective of cervical HPV infection status [5-7,9,10,20,21], our analysis was restricted to women with detectable cervical HPV infection and enabled us to focus on factors associated with seropositivity given prevalent HPV infection, also our study included a large population of young women infected with HPV-16/18, this group is of particular interest since the majority of HPV infections are acquired shortly after sexual debut.

The main finding is the observation that factors associated with sustained HPV exposure (elevated HPV viral load or cytological evidence of HPV infection), were positively associated with anti-HPV16 seropositivity. We also observed that lifetime number of sexual partners and possibly frequency of sexual intercourse were associated with anti-HPV16 seropositivity. To the extent that these factors reflects increased amount of viral exposure among infected women, this finding might also corroborate the association between viral load and seropositivity.

We also observed a positive association between current hormonal contraceptive use (oral or injectable contraceptives) and anti-HPV16 seropositivity. The effect of current oral contraceptive use on seropositivity was also observed for anti-HPV18 antibodies. We initially hypothesized that this finding could reflect increased viral load among hormonal contraceptive users given that previous studies suggested that HPV contains hormonal responsive elements and that hormonal exposure may increase viral replication[18]. However, we saw no evidence that HC2 viral load was higher among current users of hormonal contraceptives, suggesting that alternative explanations are needed to explain the association we observed between current hormonal contraceptive use and anti-HPV seropositivity. One explanation could be that hormonal factors impact the immune response directly, and thus help regulate antibody production in response to HPV infection [22,23]. Therefore, we did observe suggestive, but not statistically significant, evidence of increasing anti-HPV16 and anti-HPV18 seropositivity with increasing numbers of pregnancies.

The main limitation of our study is its cross-sectional nature. Since HPV DNA infection and anti-HPV antibodies were assessed at the same timepoint, we cannot determine the amount of time each woman had been infected with HPV-16 and/or HPV-18 at the time of DNA and serum sample collection. Studies that have evaluated time between HPV infection and seroconversion have suggested that, on average, antibody seroconversion occurs 8-12 months after infection, although these studies did not sample frequently enough [1,2]. We attempted to account for time since infection by evaluating sexual behavior variables that we believe are correlates of time since exposure/infection, such as time since sexual debut (under the assumption that HPV infection is common and that exposure/infection typically occurs proximal to sexual debut) and time with most recent partner (under the assumption that new exposures/infections are likely to occur at the start of a new relationship). While our results did suggest that seropositivity was associated with longer time with the most recent partner the effect was not statistically significant.

Overall, detection of anti-HPV16 and anti-HPV18 antibodies was observed among 63.0% and 57.5% of women infected with HPV-16 or HPV-18, respectively; this seropositivity is consistent with that reported by other investigators that about half of HPV-16 or HPV-18 DNA positive women are seropositive [7,10,24].

Despite similar rates of anti-HPV16 and anti-HPV18 seropositivity, our findings for determinants of anti-HPV18 seropositivity were less clear. The only significant predictor of anti-HPV18 seropositivity among HPV-18 infected women was current oral contraceptive use. Possible explanations include differences in the performance of the ELISA assays designed to measure antibodies against HPV-16 and HPV-18, lower power for the HPV-18 analysis since the number of HPV-18 infected women (n=179) was smaller than that of HPV-16 infected women (n=484), or true biological differences resultant from differences in patterns/location of infections caused by these two viruses. The later statement is supported by reports that HPV-18 infections are often under-represented in precancers, and that they are preferentially associated with the development of cervical adenocarcinomas that often arise deep in the endocervical canal [25,26].

## Conclusions

Our evaluation of the prevalence and determinants of anti-HPV16 and anti-HPV18 seropositivity among women with concurrent cervical infection with HPV-16 and/or HPV-18 shows that over half of HPV-16/18 infected women had detectable levels of antibodies to the HPV type with which they were infected. Factors associated with sustained HPV exposure were predictive of seropositivity (including cytology and viral load measures) as was current hormonal contraceptive use, suggesting a possible effect of hormones on immune responses to HPV. Findings were clearer for HPV-16 than for HPV-18, suggesting the need for additional studies to understand whether these differences are biologically driven or resultant from study design or assay performance. Longitudinal studies that evaluate incidently detected HPV infections and follow these infections to determine whether seroconversion occurs and their determinants are needed.

## Competing interests

Wim Quint and Leen-Jan van Doorn are employees of DDL Diagnostic Laboratory; Catherine Bougelet is an employee of GSK Biologicals. None of the authors have any potential conflicts of interest to report.

## Authors' contributions

All authors contributed to and approved the final version of the manuscript. CP, CB and AH performed the analysis of data and wrote the first draft of the manuscript. AC, RH, SW, DS, MSc and AH designed the study. CP, AC, RH, PG, SJ, AH were responsible for the coordination of the study and recruitment of the participants. LJVD and WQ were responsible for the HPV DNA testing. CB was responsible for the testing of HPV-16/18 antibodies by ELISA. MSa, SC participated in the analysis of data. MH carried out the cytological interpretations.

## Pre-publication history

The pre-publication history for this paper can be accessed here:

http://www.biomedcentral.com/1471-2334/10/238/prepub
